# A Randomized Crossover Study to Evaluate Recipe Acceptability in Breastfeeding Mothers and Young Children in India Targeted for a Multiple Biofortified Food Crop Intervention

**DOI:** 10.1177/0379572119855588

**Published:** 2019-07-30

**Authors:** Bryan M. Gannon, Varsha Thakker, Vincent S. Bonam, Jere D. Haas, Wesley Bonam, Julia L. Finkelstein, Shobha A. Udipi, Saurabh Mehta

**Affiliations:** 1 Cornell University, Ithaca, NY, USA; 2 Institute for Nutritional Sciences, Global Health, and Technology (INSiGHT), Cornell University, Ithaca, NY, USA; 3 SNDT Women’s University, Mumbai, Maharashtra, India; 4 Kasturba Health Society, Medical Research Centre, Mumbai, Maharashtra, India; 5 Arogyavaram Medical Centre, Madanapalle, Andhra Pradesh, India

**Keywords:** anthropometry, biofortification, complementary feeding, infant, stunting, wasting

## Abstract

**Background:**

A multiple biofortified food crop trial targeting iron, zinc, and vitamin A deficiencies among young children and their breastfeeding mothers is planned in India.

**Objective:**

To determine the acceptability of recipes prepared with control and biofortified pearl millet, wheat, lentils, and sweet potato.

**Methods:**

Children (6-24 months) and their mothers were enrolled as pairs (n = 52). Weight and height/length were determined. Mothers and children were separately, individually randomized in a crossover design to control or biofortified recipes. Children’s 3-day intake was measured per recipe and crop variety. For mothers, a 9-point hedonic scale evaluated color, odor, taste, and overall acceptability.

**Results:**

Children’s mean (SD) length-/height-for-age *Z*-score was −1.2 (1.7), with 27% < −2 (stunted). Mean weight-for-length *Z*-score was −0.6 (1.2) with 9.6% < −2 (wasted). Mother’s body mass index showed 17% <18.5 and 38% >25. There was no difference in the children’s intake of biofortified versus control varieties of any recipe (*P* ≥ .22); overall median daily intake was 75 g (Q1: 61, Q3: 100). Mother’s hedonic scores for color, odor, taste, or overall acceptability did not demonstrate any notable differences (*P* ≥ .23 for overall acceptability); combined median overall acceptability score was 8.5 (Q1: 8.0, Q3: 9.0).

**Conclusions:**

Recipes were consumed readily, were rated as highly acceptable, and did not show any differences between biofortified and control varieties.

## Introduction

The burden of malnutrition resulting from inadequate intakes of key micronutrients, particularly iron, zinc, and vitamin A, contributes to reduced growth, susceptibility to infection, reduced cognitive performance, and substantial morbidity and mortality globally.^1-3^ Biofortification, improving the nutritional quality of food crops through agronomic practices, conventional plant breeding, or modern biotechnology, is a promising strategy to address inadequate micronutrient intakes and improve health outcomes by enhancing nutrient content or bioavailability in commonly consumed food crops,^4^ which has received considerable research interest^5^ and international attention.^6^ Numerous crops have been biofortified with various nutrients, with the most common being iron, zinc, and provitamin A. Previous studies have demonstrated efficacy of these crops, but have thus far typically focused on single crop, single micronutrient interventions, including high iron beans and pearl millet,^7,8^ high zinc maize,^9^ high provitamin A maize,^10^ and high iron rice.^11^ Further, studies have not evaluated the impact of biofortified crops fed to mother–child pairs in the complementary feeding period.

A multiple biofortified food crop trial is underway in rural India (Clinicaltrials.gov NCT02648893). The study will include young children in the complementary feeding period (6-24 months) and their mothers, many of whom are expected to be breastfeeding their children. Two intervention arms will compare recipes prepared with biofortified crops and recipes prepared with control crops with primary outcomes of growth and indicators of malnutrition including stunting, wasting, and blood biomarkers. Recipes included in the treatment need to be suitable to children throughout the complementary feeding period as well as acceptable among participants. Further, biofortified crops can have sensory properties that may differ from conventional crops, such as the color difference between orange and white sweet potato.

The goal of this acceptability study was to determine acceptability of foods made with biofortified or control crops in young children and their mothers. This is both to determine overall recipe acceptability and between variety acceptability to confirm sufficient intervention delivery without difference by crop variety to subsequently assess multiple biofortified crops for health-related outcomes.

## Materials and Methods

### Crops

Crops were selected for inclusion based on local consumption and/or production as well as the existence of biofortified varieties containing higher concentrations of micronutrients, including high zinc wheat, high iron and zinc pearl millet, high iron lentils, and high provitamin A sweet potato. Wheat and pearl millet were grown with oversight by the International Crops Research Institute for the Semi-Arid Tropics and milled into whole grain flour. Wheat varieties used were biofortified: BHU-6, and control: HD2967. Pearl millet varieties used were biofortified: Dhanshakti, and control: DG9444. Pearl millet was used whole or milled as whole grain flour. Lentils were grown with oversight by the International Center for Agricultural Research in the Dry Areas. Lentil varieties used were biofortified: Pusa Vaibhav, control: Moitree. Lentils were dehulled before use. Sweet potato was grown with oversight by the International Potato Center (CIP). Sweet potato varieties were biofortified: Kamalasundrai, and control: white local variety. Sweet potato was washed, steamed until soft (20-30 minutes), peeled, pureed, and frozen at −18°C until use. Wheat, pearl millet, and lentils were stored in a commercial climate-controlled storage facility (53%–56% humidity, temperature 6°C-14°C).

### Recipe Development and Preparation

Recipes were developed at SNDT Women’s University and Kasturba Health Society–Medical Research Centre in Mumbai in consultation with local study staff. Recipes inclusion strategies were (1) traditionally consumed recipes already using crops of interest, (2) traditional recipes modified by adding or substituting ingredients (eg, sweet potato in chutney or pearl millet instead of rice), and (3) novel recipes. Local staff was trained in preparation of the standardized recipes. Yield was calculated by weighing prepared food and divided by weight of all raw recipe components. Recipes used, preparation, and yields are provided in Table 1. Within recipe, the only difference between arms was inclusion of biofortified or control crops.

**Table 1.** Recipe Description and Methodology.

No Recipe and Yield	Recipe and Yield	Description, Ingredients, Weights (per 100 g), and Preparation Protocol
Savory		
1	Pearl millet idli Yield: 102%	Steamed cake Ingredients: Coarsely ground pearl millet (57 g), curd (29 g), water (14 g), sodium bicarbonate (0.1 g), salt (1.0 g) Directions: Mix together coarsely ground pearl millet, curd, and water. Allow to sit for 1 hour. Add salt and sodium bicarbonate and mix into batter. Portion batter into idli mold and cook with steam for 20 to 30 minutes until firm. Serve warm.
2	Lentil sambar Yield: 94%	Side relish Ingredients: Dry lentil (17 g), onion (4.5 g), tomato (7.6 g), green chilli (0.3 g), coriander leaves (0.6 g), tamarind (1.1 g), oil (1.1 g), garlic (0.4 g), curry leaves (1.0 g), water (67 g), sambar powder (0.5 g) Directions: Cook lentils in water until very soft, mash thoroughly. Soak tamarind in water for 15 minutes and extract thick juice. Peel onions and chop vegetables. Heat a pan with oil. Sautee curry leaves and onions. Add remaining vegetables and sauté for 4 minutes. Add tamarind juice and cook 3 to 5 minutes. Add mashed lentils and stir. Add sambar powder, salt to taste, and cook 5 more minutes. Add coriander leaves and serve warm.
3	Sweet potato chutney Yield: 97%	Side relish Ingredients: Sweet potato puree (71 g), fresh coconut (18 g), coriander leaves (2.1 g), green chilli (1.4 g), cumin seed (0.7 g), curry leaves (4 leaves), oil (1.8 g), mustard seed (0.7 g), asafetida powder (0.4 g) Directions: Heat oil in pan, add spices, and sautee for 10 minutes. Add sweet potato and coconut, mix, and cook for 2 minutes. Serve room temperature.
4	Pearl millet mudde (ball) Yield: 90%	Ball cake Ingredients: Pearl millet (33 g), water (65 g), oil (1.6 g), salt (0.3 g) Directions: Dry roast pearl millet flour. Boil water and add oil and salt. Add roasted flour slowly and stir continuously, once mixed, allow to sit covered off heat for 5 minutes. Roll into balls and serve warm.
5	Pearl millet and sweet potato Pulao Yield: 109%	Pilaf with vegetables Ingredients: Coarsely ground pearl millet flour (26 g), water (52 g), oil (5.2 g), onion (1.3 g), green peas (1.3 g), green beans (1.3 g), carrot (1.3 g), capsicum (0.5 g), sweet potato (8 g), green chilli (0.3 g), mustard seeds (0.4 g), garlic paste (0.5 g), cinnamon (0.1 g), cardamom (0.1 g), ground peppercorn (0.3 g), curry leaves (0.5 g), coriander leaves (0.5 g) Directions: Wash and soak pearl millet 1 hour. Strain, wash, and boil with measured water approximately 30 minutes until soft. Sautá vegetables and spices in oil for about 15 minutes, add to pearl millet, mix well, and cook covered for 5 to 10 more minutes. Garnish with chopped coriander leaves.
6	Sweet potato rasam Yield: 97%	Thick soup Ingredients: Sweet potato puree (29 g), tomato puree (20 g), water (49 g), coriander leaves (0.1 g), curry leaves (0.1 g), rasam powder (1 g), salt (1 g) Directions: Add sweet potato and tomato to pot, cook over medium heat for 10 minutes. Add remaining ingredients and cook for 5 more minutes. Serve room temperature.
Sweet		
7	Wheat and sweet potato kesari Yield: 81%	Sweet porridge/pudding Ingredients: Wheat flour (13 g), sweet potato puree (13 g), sugar (13 g), ghee (10 g), cardamom powder (0.5 g), hot water (51 g) Directions: In pan over low flame, heat the ghee, add wheat flour, and roast until color changes, approximately 10 to 15 minutes. Add sweet potato puree, stir constantly, and cook for 5 minutes. If necessary adjust consistency with water. Stir in sugar and cardamom. Serve warm.
8	earl millet porridge Yield: 77%	Sweet pudding Ingredients: Pearl millet flour (8 g), hot water (78 g), sugar (10 g), ghee (3 g), cinnamon (0.5 g), cardamom (0.5 g) Directions: In pan over medium flame, heat the ghee, add pearl millet flour, and roast until aroma is present, 5 to 7 minutes. Add hot water and sugar and stir. Cook until desired consistency is reached and serve warm.
9	Wheat porridge Yield: 95%	Sweet pudding Ingredients: Wheat flour (14 g), hot water (69 g), sugar powder (14 g), ghee (2 g), cinnamon (0.5 g), cardamom (0.5 g) Directions: In pan over medium flame, heat the ghee, add wheat flour, and roast until aroma is present, 5 to 7 minutes. Add hot water and sugar and stir. Cook until desired consistency is reached and serve warm.
10	Sweet potato thick shake Yield: 100%	Thick drink Ingredients: Sweet potato puree (44.3 g), milk (44.3 g), sugar (11 g), cinnamon (0.1 g), cardamom (0.1 g) Directions: Blend all ingredients well, and serve chilled/cold.
11	Sweet potato barfi Yield: 93%	Sweet thick bar Ingredients: Sweet potato puree (49 g), ground peanuts (16 g), sugar (16 g), ghee (2 g), cardamom powder (0.3 g), cinnamon powder (0.3 g), water (16 g) Directions: In pan on medium heat, boil sugar and water into a thick syrup. Add remaining ingredients and stir continuously until mixture is thick. Spread onto a greased plate, allow to cool, and cut for serving. Serve room temperature.
12	Payasam Yield: 99%	Sweet thick soup Ingredients: Sweet potato puree (30 g), milk (61 g), jaggery (9 g), cardamom (0.1 g) Directions: Boil milk and allow it to cool. Heat sweet potato and jaggery over medium flame and stir until mixed. Mix in milk and cardamom, stir, and allow to set. Serve room temperature.
13	Sarbat Yield 100%	Cool drink Ingredients: Sweet potato puree (22 g), hot water (67 g), sugar (9 g), lemon juice (1 g), roasted cumin powder (0.3 g), salt (0.3 g) Directions: Boil water, puree all ingredients, and serve chilled.
14	Wheat, lentil, and sweet potato puran poli Yield: 70%	Stuffed flatbread Ingredients: Wheat flour (16 g), refined all-purpose wheat flour (4 g), water for dough (12 g), water to cook lentils (25 g), lentils (12 g), sweet potato puree (19 g), sugar (8 g), groundnut powder (0.5 g), cardamom powder (0.3 g), oil (1 g), salt (0.2 g) Directions: To make filling, rinse and boil lentils until soft. Mix in sweet potato, sugar, groundnut, cinnamon, cardamom, and cook until mixture is semisoft, approximately 15 minutes. Keep filling aside and let cool before handling. For outer flatbread, make dough from wheat flour, all-purpose wheat flour, water, and oil. Knead until soft. Make dough ball of approximately 60 g, roll out, place approximately 90 g filling inside, and fold dough around filling. Shallow fry about 2 minutes each side. Cut as needed for serving.

### Participants and Setting

This study was conducted in 2 feeding centers in a rural setting near Madanapalle, Andhra Pradesh. Inclusion criteria were children 6- to 24-month-old as reported at enrollment, mothers of included children, and indication of willingness to return to the feeding center on subsequent days. Exclusion criteria were any dietary allergies, currently diagnosed with malaria or dengue, ever diagnosed with HIV or tuberculosis, or severe malnutrition (ie, weight-for-length *Z*-score [WLZ] < −3) determined using World Health Organization (WHO) field tables.

### Study Design

*Design, randomization, allocation, and masking*. The study design was based on a modified version of an acceptability study in young children and their mothers.^12^ This included a multiple day evaluation period, a return to usual diet, and a crossover to the different variety to be evaluated.

**Figure 1.**
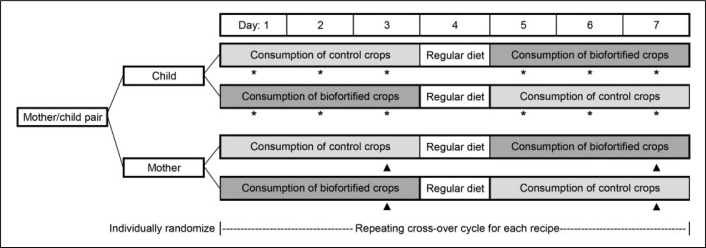
Study design for mother–child pairs separately, individually randomized to a crossover evaluation of biofortified and control crops. For each recipe, participants evaluated the recipe made from either control or biofortified crops for 3 days, had a 1 day break without feeding, then switched to the opposite crop variety. Child’s food intake was weighed each day (*). Mother’s hedonic scale was evaluated on the last day of each recipe variety (days 3 and 7, ▲). This cycle repeated for each recipe evaluated.

This study was conducted from December 2017 through April 2018. For each recipe tested, a 2 × 2 crossover design was used (Figure 1). Children and mothers (n = 52 pairs) were separately, independently randomized by individual to a sequence to reduce maternal influence on child feeding. The sequence indicated either biofortified or control variety for each recipe before crossing over to the other variety. Once randomized, each participant followed the same sequence for each recipe. Two randomization sequences (one each for children and mothers) were generated by computer. Allocation was done with 2 sets of sequentially numbered opaque envelopes opened once a participant was enrolled. Envelopes were prepared by staff not involved with participant recruitment. Recipes were coded by food type (control and biofortified) and not revealed to participants.

*Feeding*. Children consumed one recipe prepared from one type of crop (ie, control or biofortified) for 3 consecutive days, had a 1 day break where participants did not come to the feeding center, and then crossed over to the same recipe prepared with the other type of crop for 3 consecutive days (Figure 1). Mothers sampled 1 to 3 recipes per cycle remaining within crop variety. For each recipe, this cycle was repeated and participants remained on the same sequence. Eight recipe cycles were conducted at each feeding center. Some recipes were only tested at one feeding center, and some were tested at both feeding centers to optimize number of recipes evaluated and also compare some recipes between feeding centers. Participants only attended one feeding center. On each day of feeding, mothers and children arrived at the feeding center as convenient for the participants within a specified time window. The feeding center included a dedicated room where participants ate together to simulate conditions for the main trial.

The child was fed first; for each children’s recipe, a weighed serving was provided, and children were given additional weighed servings as desired (ie, ad libitum). For the child’s feeding episode, mothers were instructed to give children enough opportunity to eat, finishing when the child refused food 3 times. After the child’s feeding episode, the weight of remaining food was recorded. Eleven recipes were evaluated in children. Two recipes were consumed as recipe components together per standard feeding practice (idly and sambar; pulao and sambar), and remaining food was weighed together. Time at start and stop of child’s feed was recorded to determine feeding duration.

Mothers were provided food after the children were finished eating to prevent influence on the child’s eating behavior. Fourteen recipe components were evaluated by mothers as some recipes were provided as specifically appropriate to mothers. Mothers were asked not to share food among participants. On the third day of each crossover sequence, mothers separately rated the color, odor, taste, and overall acceptability of each recipe on a 9-point hedonic scale with a questionnaire administered by research staff.

*Equipment*. Length and height were measured to the nearest 0.1 cm using ShorrBoards (Weight and Measure, LLC, Olney, Maryland). Participant weights were measured to the nearest 0.1 kg with a mother/child scale (Seca, Hamburg, Germany). Digital scales for weighing food during preparation, serving, and consumption were SGS certified, and food measurements were recorded to the nearest 1 g.

### Ethical Approval

Arogyavaram Medical Centre Institutional Ethics Committee approved the study protocol in accordance with the Declaration of Helsinki. The nature of the study was explained to mothers, and all provided signed, written informed consent.

### Data Analysis

Height or length was taken in triplicate and the mean was calculated. Weights were obtained in singlet. Anthropometric *Z*-scores were calculated (1) by WHO field tables to determine study inclusion, and (2) by WHO Anthro (3.2.2, WHO)^13^ for reporting.

Data are reported as mean (SD), median (Q1, Q3), predicted population margins (least squares mean [standard error, SE]), or percentage. Statistical analysis was done in SAS v9.4 (University Edition; SAS institute, Cary, North Carolina). Mixed models were used for child’s recipe intake and mother’s hedonic scale. For child’s recipe intake, the model initially included fixed effects of crop variety, age, sex, sequence, with random effects of child and child by crop variety. For mother’s hedonic scale, the model initially included fixed effects of crop variety and sequence and a random effect of mother. For recipes evaluated at both feeding centers, an effect of site and a group by site interaction were also tested. Residual normality was confirmed, and if non-normal, variables were rank transformed before analysis. *P* < .05 was considered significant; no adjustment was made for multiple comparisons as a low threshold to detect potential crop differences was desired.

## Results

### Participants, Anthropometry, and Compliance

Fifty-two pairs of children and mothers were enrolled. Baseline participant characteristics and anthropometry are presented (Table 2). Twelve participant pairs attended for less than 6 days. Of the remaining, median (Q1, Q3) daily attendance compliance was 62.8% (44.1%, 83.5%).

**Table 2.** Participant Characteristics and Anthropometry.^a^

Children	
Female (%)	52
Age (months)	14.3 (5.6)
Weight (kg)	8.3 (1.4)
Length (cm)	72.2 (5.2)
LAZ	–1.2 (1.7)
Stunting, % LAZ < – 2	26.9
WLZ	–0.62 (1.2)
Wasting, % WLZ < – 2	9.6
Mothers	
Age (years)	24.2 (3.6)
Weight (kg)	55.6 (12.1)
Height (cm)	154 (5.8)
BMI (kg/m2)	23.3 (4.7)
BMI (% < 18.5)	17.3
BMI (% > 25)	38.5

Abbreviations: BMI, body mass index; LAZ, length-for-age Z-score; WLZ, weight-for-length Z-score.

a Data are means (SD) or %; n ¼52.

### Child Food Intake

No significant differences in child’s food intake were observed between biofortified and control varieties for any recipe (Table 3). There was also no effect of child’s sex on any recipe. The effect of age was only significant for the recipe kesari, where each additional month of age corresponded to an additional intake of 1.75 g per feeding episode. Sequence was not significant for any recipe (*P* ≥ .20). Child feeding duration did not differ between varieties for any recipe (*P* ≥ .25), and mean (SD) ranged from 15.4 (6.3) minutes for thick shake to 26.1 (6.0) minutes for pulao.

**Table 3:** Children’s Intake of Recipes Made From Biofortified or Control Crops by Day.^a^

Recipe	Biofortified Intake (g) Median (Q1, Q3) [n]	Control Intake (g) Median (Q1, Q3) [n]
Barfi^b^	75 (75, 75) [37]	75 (74, 75) [44]
Idly and sambar^b^	100 (100, 100) [34]	100 (98, 100) [40]
Kesari^b,c^	70 (44, 80) [81]	70 (44, 70) [72]
Payasam	100 (62.5, 125) [24]	105 (75, 125) [23]
Pearl millet balls and rasam	125 (115, 125) [66]	125 (123, 125) [73]
Pearl millet porridge	70 (57, 70) [45]	64 (48, 70) [46]
Poli	70.5 (60, 75) [84]	68 (62, 75) [81]
Pulao and sambar	75 (55, 80) [26]	75 (60, 95) [27]
Sharbat	100 (100, 100) [70]	100 (100, 100) [70]
Thick shake	61.5 (41, 70) [80]	63.5 (35, 74.5) [92]
Wheat flour porridge	49 (37, 63) [22]	50 (39, 66.5) [24]

^a^ Data were analyzed by a mixed model accounting for repeated measures for child and child by crop variety, adjusting for age and sex. For all recipes, crop variety and sex were not significant predictors of intake (*P* ≥ .22 and .13, respectively).

^b^ Rank transformed for statistical analysis.

^c^ Kesari recipe had a significant effect of age in months (b ¼1.75, standard error ¼0.62, *P* ¼.0060), no other recipes had a significant effect of age (*P* ≥ .06).

Children’s intake of 5 recipes was evaluated at both centers. None had a significant site by crop variety interaction (*P* ≥ .22). Two (poli and sharbat) had no difference in intake by site (*P* ≥ .39). Two had greater intake at site 1 (least squares mean [SE] for kesari: 76.5 [3.0] vs 46.3 [3.8] and pearl millet balls with rasam: 122.0 [2.3] vs 112.1 [3.1] g; *P* ≤ .011). One recipe (thick shake) had greater intake at site 2 (75.4 [7.0] vs 49.9 [3.6] g; *P* = .0019).

### Mother’s Acceptability

Overall acceptability of hedonic scale rankings did not differ significantly between biofortified and control varieties for any recipe (*P* ≥ .23; Table 4). All medians were ≥ 8.0 indicating high acceptability. There were also no significant differences between biofortified and control recipes for color (Table 5, *P* ≥ .11) or odor (Table 5, *P* ≥ .11). Control lentil sambar had a significantly higher taste in the hedonic scale compared to biofortified; however, the biofortified variety was still very acceptable (median: 8.8). Remaining taste hedonic scores did not differ between crop varieties (Table 5, *P* ≥ .09).

**Table 4.** Mother’s Hedonic Scale: Overall Acceptability.^a^

Recipe	Biofortified Median (Q1, Q3) [n]	Control Median (Q1, Q3) [n]
Barfi	9.0 (9.0, 9.0) [12]	9.0 (8.0, 9.0) [17]
Chutney	8.0 (7.5, 8.7) [32]	8.0 (8.0, 8.5) [29]
Idly	8.5 (8.0, 8.8) [38]	8.5 (8.0, 8.8) [36]
Kesari	9.0 (8.0, 9.0) [25]	9.0 (9.0, 9.0) [21]
Payaasam	9.0 (9.0, 9.0) [9]	9.0 (9.0, 9.0) [9]
Pearl millet balls	8.0 (8.0, 9.0) [21]	8.0 (7.0, 8.0) [24]
Pearl millet porridge	9.0 (9.0, 9.0) [15]	9.0 (9.0, 9.0) [12]
Pulao	8.0 (7.0, 8.0) [9]	8.0 (2.0, 8.0) [11]
Poli	9.0 (8.5, 9.0) [25]	9.0 (9.0, 9.0) [25]
Rasam	8.0 (7.5, 9.0) [22]	8.0 (7.5, 8.0) [24]
Sambar	8.5 (8.3, 9.0) [24]	9.0 (8.5, 9.0) [18]
Sharbat	8.0 (8.0, 9.0) [19]	8.0 (8.0, 9.0) [17]
Thick shake	9.0 (8.0, 9.0) [27]	8.0 (8.0, 9.0) [23]
Wheat flour porridge	8.0 (8.0, 8.5) [8]	8.0 (8.0, 9.0) [8]

a Data were analyzed by a mixed model accounting for repeated measures for mother; sequence was not significant for any recipe and not included in the final model. All P values reflecting effect of variety on hedonic score were F020 ≥.12.

**Table 5.** Mother’s Hedonic Scale: Color, Odor, and Taste.^a^

Recipe [Biofortified n, Control n]	Color		Odor		Taste	
	Biofortified	Control	Biofortified	Control	Biofortified	Control
Barfi [12, 17]	9.0 (9.0, 9.0)	9.0 (8.0, 9.0)	9.0 (9.0, 9.0)	9.0 (8.0, 9.0)	9.0 (9.0, 9.0)	9.0 (8.0, 9.0)
Chutney [32, 29]	8.0 (7.6, 8.7)	8.0 (8.0, 8.5)	8.0 (7.4, 8.6)	8.0 (7.8, 8.5)	8.0 (7.5, 8.8)	8.0 (8.0, 8.5)
Idly [38, 36]	8.3 (8.0, 8.8)	8.0 (8.0, 8.5)	8.2 (7.8, 8.5)	8.0 (8.0, 8.5)	8.5 (8.0, 8.8)	8.5 (8.0, 9.0)
Kesari [25, 21]	9.0 (8.0, 9.0)	9.0 (8.0, 9.0)	9.0 (8.0, 8.0)	9.0 (8.0, 9.0)	9.0 (8.0, 9.0)	9.0 (9.0, 9.0)
Payaasam [9, 9]	9.0 (9.0, 9.0)	9.0 (9.0, 9.0)	9.0 (9.0, 9.0)	9.0 (9.0, 9.0)	9.0 (9.0, 9.0)	9.0 (9.0, 9.0)
Pearl millet balls [21, 24]	8.0 (7.0, 9.0)	8.0 (7.5, 8.0)	8.0 (8.0, 9.0)	8.0 (7.0, 8.0)	8.0 (7.0, 9.0)	8.0 (7.0, 8.0)
Pearl millet porridge [15, 12]	9.0 (9.0, 9.0)	9.0 (9.0, 9.0)	9.0 (9.0, 9.0)	9.0 (9.0, 9.0)	9.0 (9.0, 9.0)	9.0 (9.0, 9.0)
Pulao [9, 11]	8.0 (7.0, 8.0)	8.0 (2.0, 8.0)	8.0 (7.0, 8.0)	8.0 (2.0, 8.0)	8.0 (7.0, 8.0)	8.0 (2.0, 8.0)
Poli [25, 25]	9.0 (8.5, 9.0)	9.0 (8.5, 9.0)	9.0 (8.5, 9.0)	9.0 (8.5, 9.0)	9.0 (8.5, 9.0)	9.0 (9.0, 9.0)
Rasam [22, 24]	8.0 (8.0, 9.0)	8.0 (8.0, 8.0)	8.0 (8.0, 9.0)	8.0 (8.0, 8.0)	8.0 (8.0, 9.0)	8.0 (7.8, 8.0)
Sambar [24, 18]	8.5 (8.0, 9.0)	9.0 (8.5, 9.0)	8.5 (8.0, 9.0)	9.0 (8.5, 9.0)	8.8 (8.5, 9.0)^b^	9.0 (9.0, 9.0)
Sharbat [19, 17]	8.0 (8.0, 9.0)	8.0 (8.0, 9.0)	8.0 (8.0, 9.0)	8.0 (8.0, 9.0)	8.0 (8.0, 9.0)	8.0 (8.0, 9.0)
Thick shake [27, 23]	8.0 (8.0, 9.0)	8.0 (8.0, 9.0)	8.0 (7.0, 9.0)	8.0 (7.0, 9.0)	8.0 (8.0, 9.0)	8.0 (8.0, 9.0)
Wheat flour porridge [8, 8]	8.0 (8.0, 8.5)	8.0 (8.0, 8.0)	7.5 (7.0, 8.5)	8.0 (8.0, 8.0)	8.0 (8.0, 8.5)	8.5 (8.0, 9.0)

^a^ Data are median (Q1, Q3). Scores without a common superscript differ by variety by attribute, *P* < .05, a > b.

^b^ Taste attribute for rasam recipe differed between biofortified and control varieties, *P* < .05. No other recipe differed significantly by crop variety for any attribute.

Mother’s hedonic scale of 8 recipes was evaluated at both centers. Two recipes had higher overall acceptability ratings at center 1 (least squares mean [SE] kesari: 8.9 [0.1] vs 8.2 [0.1] and pearl millet balls: 8.2 [0.4] vs 7.0 [0.4]; *P* ≤ .049). One recipe (rasam) had a significant group by site interaction, where the biofortified variety at center 1 had higher overall acceptability (*P* ≤ .033) than other variety by site combinations, which did not differ (*P* ≥ .51). The remaining 5 recipes (chutney, idly, poli, sharbat, and thick shake) did not have a significant difference by site or a group by site interaction (*P* ≥ .20).

## Discussion

### Overall Conclusion

This pilot acceptability study determined that recipes developed for use in this trial were consumed by children, rated highly by mothers, and did not display statistically significant differences in these outcomes between control and biofortified crops. This study is novel in that multiple biofortified food crops are used in the same recipes and menu to increase intakes of multiple micronutrients, iron, zinc, and vitamin A.

### Strengths/Limitations

Strengths of this study included the individually randomized, crossover design and multiday evaluation for each recipe variety. This reduced confounding due to factors such as child age or day of week that could affect dietary intakes. Mothers and children were randomized separately, and children were fed first each day to reduce potential bias of a mother’s preference on her child’s feeding. Two feeding centers, approximately 8 km apart, were used to include geographically distinct locations. Limitations for generalizability to the main planned feeding trial include feeding only 1 meal per day compared to 3 meals in the trial, a repetitive weekly menu, and low compliance among some individuals.

Low compliance among some participants was due to initial interest and enrollment, but inability to attend subsequent days. Reasons included traveling away from the study area, having to travel too far to the study center, or recipe fatigue. The main study will address these issues by ensuring participants plan to remain in the study area before enrollment, having sufficient feeding centers to accommodate participant travel, and serving a diverse rotating menu. Occasionally, children consumed minimal food on some days, which was attributed to recently breastfeeding or lack of appetite.

### Results in Context: Anthropometry

Anthropometric indicators of malnutrition in children were high and followed similar patterns to the National Family Health Survey (NFHS) for Andhra Pradesh in rural settings.^14^ Stunting (length-for-age *Z*-score < −2) prevalence in this population was 27% compared to 33% in rural settings in NFHS. Wasting (WLZ < −2) prevalence was 10% in this population compared to 18% in rural settings in NFHS. For mothers, prevalence of body mass index (BMI) below normal (BMI < 18.5) was 17% compared to 20% from NFHS. Prevalence of overweight (BMI ≥ 25) was 39% compared to 28% in NFHS. Both under- and overnutrition were observed in this population, and this will be considered when designing a menu and serving sizes to optimize micronutrient content while balancing macronutrient intakes.

### Results in Context: Acceptability of Biofortified Crops

Numerous studies have found that biofortified crops are consumed readily and similarly to control counterparts across age ranges. A systematic review demonstrated that biofortified crops are acceptable in both urban and rural settings, when prepared with traditional or novel methods, and despite color changes accompanying some biofortified crops.^15^ Orange sweet potato has been extensively shown to be equivalent, or preferred, to white sweet potato. A study of orange sweet potato curd in adults from rural Orissa, India, demonstrated that color and texture were main drivers of consumer acceptability.^16^ In a community-based trial where women and children were given control or zinc-biofortified wheat flour to use at home in Delhi, compliance was high and similar among wheat varieties among women and children.^17^ Our research team and collaborators recently conducted an acceptability study in young children in Mumbai where they evaluated recipes prepared with biofortified and control pearl millet and found recipes acceptable, with minor differences noted in consumption or rating of recipes.^18^ In this study, we saw greater consumption and essentially no difference between biofortified and control crops, likely due to the randomized crossover design that controls for potential confounders related to food preparation or feeding on a given day.

We expected to see an effect of age on recipe consumption in children; however, only one recipe, kesari, demonstrated a significant effect of age, with consumption increasing significantly with age. The lack of difference in most recipes may be due to the fact that this was a single feeding episode considered a snack which may not have reflected differences in overall food intake throughout the day. We did not see an interaction between recipe and crop variety on consumption time, which provides further evidence that both crop varieties were consumed similarly by participants. We observed differences in child intakes or mother’s acceptability by site in some recipes, which tended to correlate by recipe; site 1 had higher children’s intake and mother’s acceptability of kesari and pearl millet balls. This could have been due to site-specific preferences or confounding related to the time of evaluation that differed by site, for example, weather affecting preferences of hot or cold foods.

## Conclusion

This pilot study has demonstrated a substantial burden of malnutrition and potential to benefit in this population. Among participants similar to those who will be targeted for the intervention trial, there was a willingness to attend a feeding center and consume recipes made from multiple crops, with no apparent difference in preference or consumption between crop varieties.

## Authors’ Note

S.M. is principal investigator and has primary responsibility for final content. J.D.H., J.L.F., and S.A.U. are investigators on the parent trial. B.M.G. and S.M. designed this research study. V.T. and S.A.U. developed recipes and trained staff in preparation. V.S.B. and W.B. oversaw fieldwork and data collection. B.M.G. analyzed data and wrote the first draft of the manuscript. All authors read and approved the final manuscript.
